# Posterior Mitral Valve Acute Infective Endocarditis Complicated With Brain Abscess and Infection-Related Glomerulonephritis: A Case Report

**DOI:** 10.7759/cureus.102782

**Published:** 2026-02-01

**Authors:** Mikunthan Mithuriha, Raja Hettiarachchi, Thamotharam Balachandran

**Affiliations:** 1 Internal Medicine, Colombo North Teaching Hospital, Ragama, LKA; 2 Medicine, National Hospital of Sri Lanka, Colombo, LKA

**Keywords:** brain abscess, infective endocarditis, mitral valve, septic emboli, staphylococcus aureus

## Abstract

We report the case of a 48-year-old previously unevaluated woman who presented with a short febrile illness with multi-organ involvement and was later diagnosed with acute methicillin-sensitive *Staphylococcus aureus* infective endocarditis of the posterior mitral valve. Her illness was complicated by septic embolization, leading to cerebral abscesses and infection-related glomerulonephritis, causing acute kidney injury. She had multiple septic teeth, which were thought to be the source of bacteremia. She was managed with multidisciplinary input involving cardiology, neurology, neurosurgery, microbiology, and oral maxillofacial surgery. Six weeks of intravenous antibiotics and removal of dental foci resulted in complete clinical and biochemical recovery. Follow-up transesophageal echocardiogram demonstrated resolution of the vegetation, and interval imaging showed a reduction in the size of the brain abscess. This case highlights the importance of early recognition of infective endocarditis and the need for multidisciplinary care in the presence of systemic complications.

## Introduction

Infective endocarditis (IE) is one of the life-threatening conditions associated with high morbidity and mortality due to systemic embolization and diffuse organ dysfunction [1]. *Staphylococcus aureus* is a common cause of acute IE and is frequently associated with rapid clinical deterioration and septic embolization, especially cerebral abscesses. Infection-related glomerulonephritis is also a recognized immune-mediated complication of IE, presenting with microscopic hematuria, proteinuria, and acute kidney injury. Early diagnosis, identification of the infectious source, and targeted antimicrobial therapy for the recommended duration are important management principles to prevent complications [2]. Here, we report a case of acute methicillin-sensitive *Staphylococcus aureus* (MSSA) IE of the posterior mitral valve, which was complicated by septic embolic (brain abscesses) and infection-related glomerulonephritis, successfully managed with patient-centered targeted medical therapy and a multidisciplinary team approach.

## Case presentation

**Figure 1:**
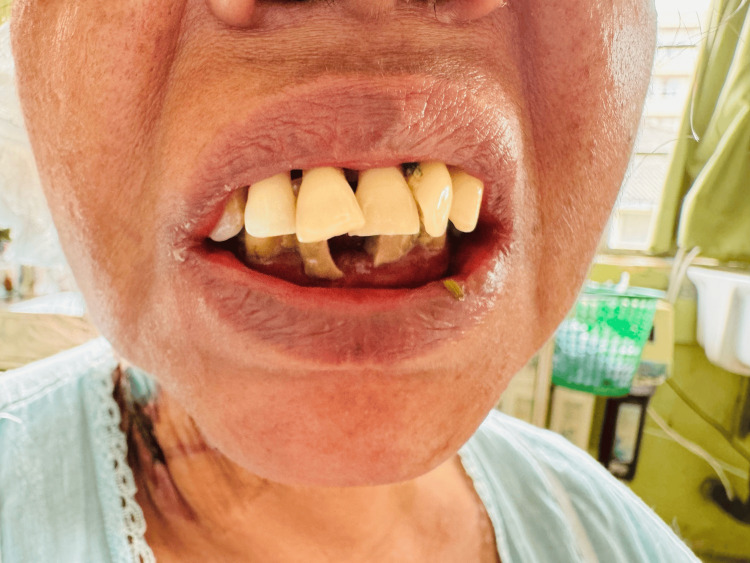
Poor oral hygiene with multiple septic teeth.

**Figure 2:**
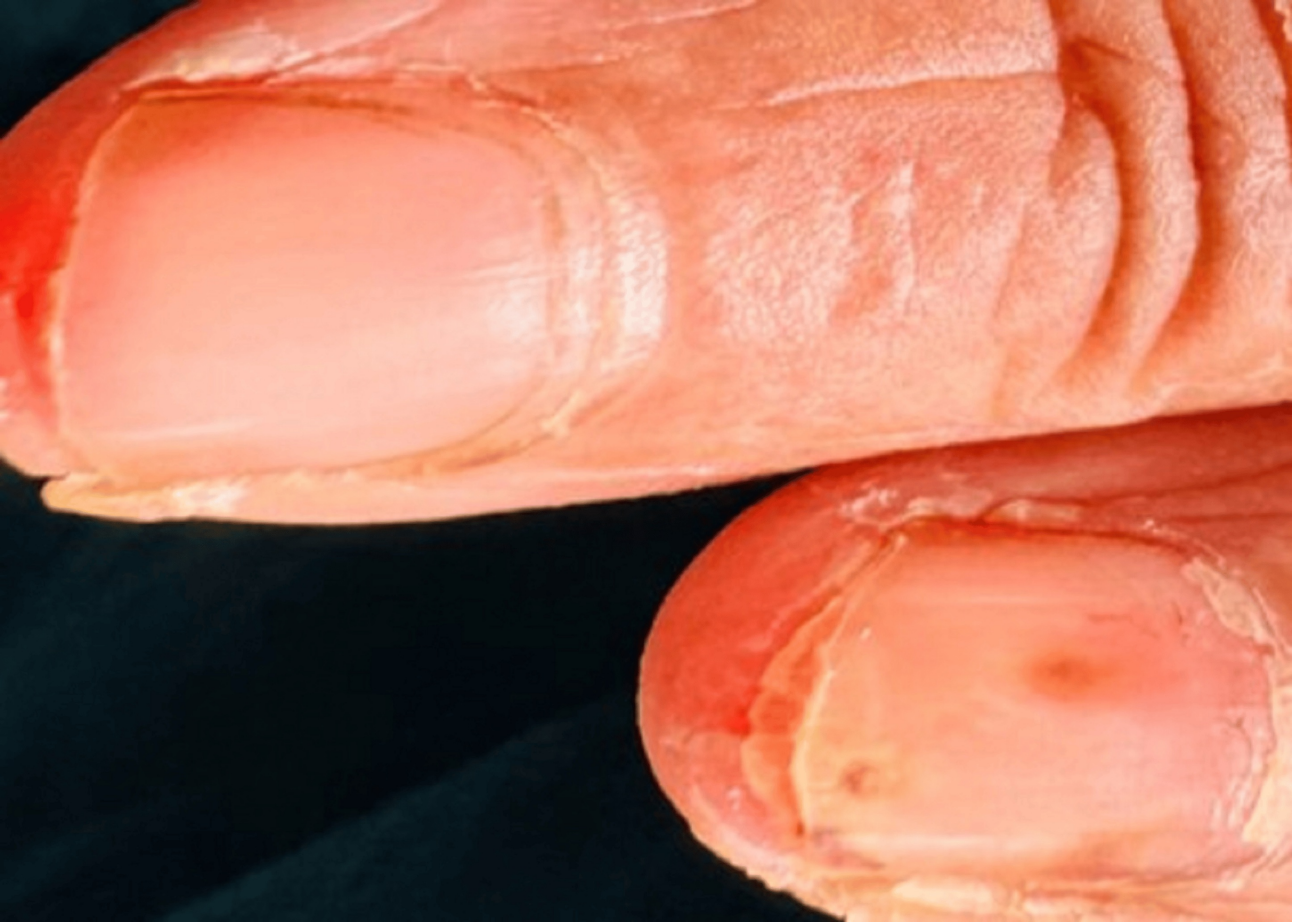
Splinter hemorrahges.

**Figure 3:**
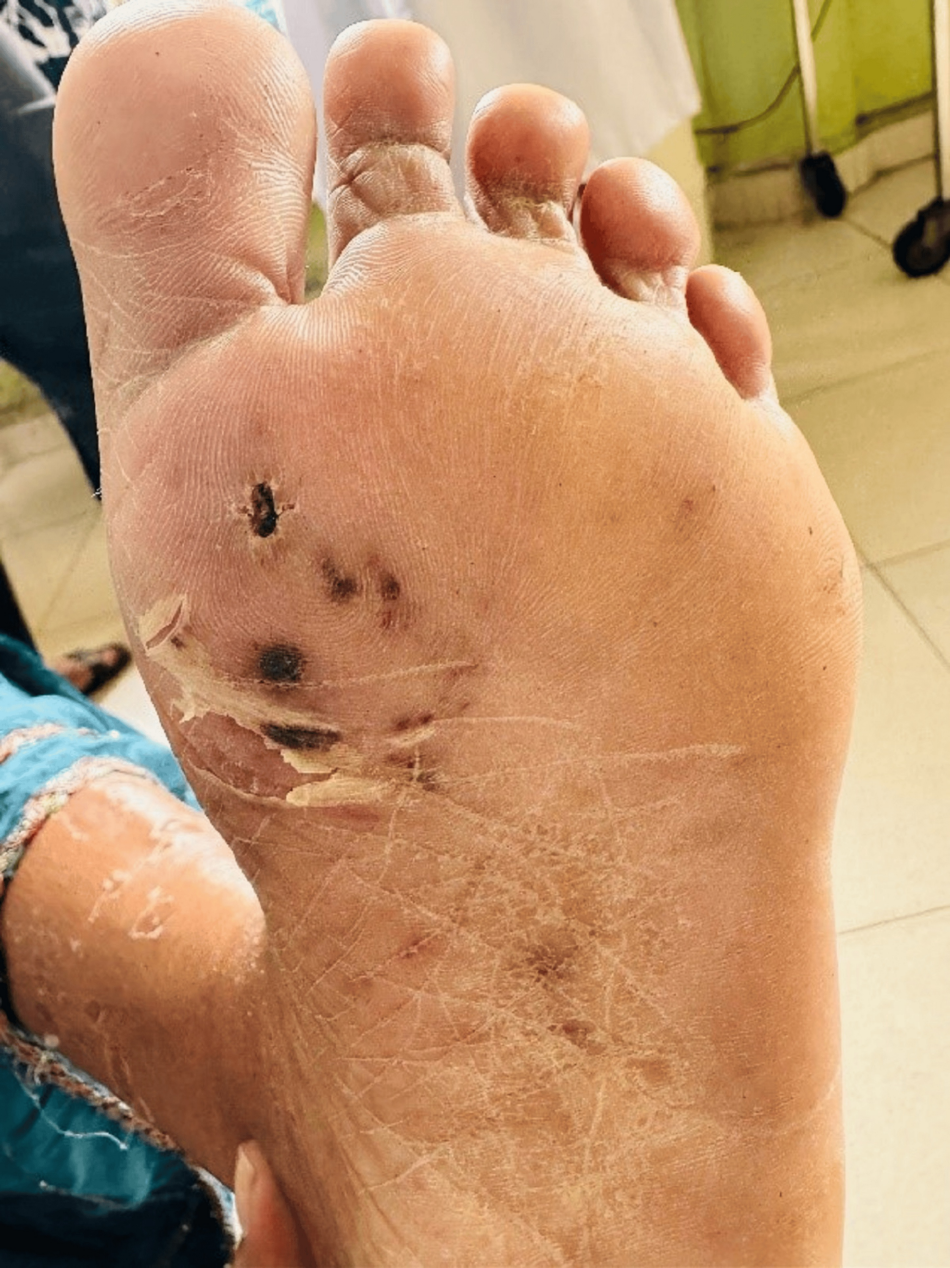
Painless plantar lesions (Janeway lesions).

**Figure 4:**
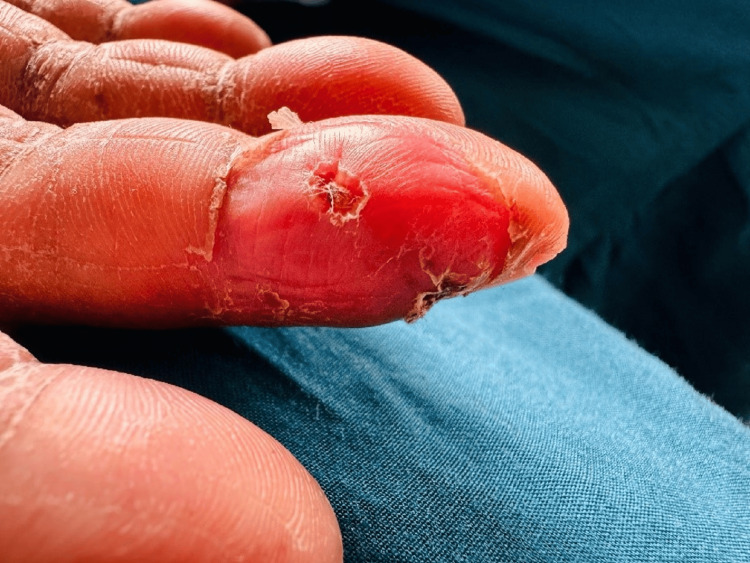
Painful palmar pulp lesion (Osler’s nodes).

A 48-year-old previously healthy woman presented with a four-day history of high-grade intermittent fever with chills and rigors and watery loose stools with frequency of four to five times daily. There was no vomiting, abdominal pain, or blood/mucus in the stools. She also reported arthralgia, myalgia, generalized weakness, and mild headache. There was no cough, dyspnea, chest pain, urinary symptoms, recent travel, intravenous drug use, or muddy-water exposure. She was a non-smoker, non-alcoholic, and a housewife. On examination, she was febrile and pale with poor oral hygiene and multiple dental caries (Figure 1). Cutaneous stigmata suggestive of IE were present: splinter hemorrhages (Figure 2), painless palmar and plantar macules (Janeway lesion) (Figure 3), and painful digital pulp lesions (Osler’s node) (Figure 4). Cardiovascular examination revealed a regular pulse (80 beats/minute); however, there were no audible murmurs. Respiratory and abdominal examinations were normal. Neurological examination showed normal fundoscopy, mild generalized hypotonia, preserved reflexes, and power of 4/5 in all limbs. During the course of ward stay, she developed sudden-onset right upper limb weakness and right upper motor neuron facial nerve palsy for which she was further evaluated.

Table 1 summarizes the investigations of a patient with severe MSSA sepsis, characterized by neutrophilic leukocytosis, markedly elevated inflammatory markers, and complicated by acute kidney injury, transaminitis, rhabdomyolysis, and sepsis-related myocardial injury. Blood cultures grew MSSA, while extensive infective, autoimmune, and cerebrospinal fluid evaluations were negative. Following treatment, there was marked clinical and biochemical improvement with normalization of organ function, recovery of blood counts, and reduction of inflammatory markers.

**Table 1:** Laboratory and blood investigations.

	On admission	After treatment	Normal range
Full blood count			
White blood cells (10^9^/L)	17.64	9.7	4.0–11.0
Neutrophils (10^9^/L)	14.14	6.3	2.0–7.0
Lymphocytes (10^9^/L)	2.17	2.1	1.0–4.0
Monocytes (10^9^/L)	1.02	1.0	0.2–0.8
Eosinophils (10^9^/L)	0.09	0.1	0.02–0.4
Basophils (10^9^/L)	0.22	0.2	0.01–0.1
Hemoglobin (g/dL)	9.5	11	12.0–15.5
Platelets (10^9^/L)	155	250	150–450
Blood picture	The blood picture shows normocytic normochromic anemia with evidence of ongoing severe bacterial infection. Thrombocytopenia could be due to infection. No evidence of MAHA		
Erythrocyte sedimentation rate (mm/hour)	115	40	0–20
C-reactive protein (mg/dL)	294	20	<5
Procalcitonin (ng/ml)	47	N/A	<0.1
Serum creatinine (µmol/L)	586	80	62–115
Blood urea (mg/dL)	184	10	7–20
Aspartate aminotransferase (U/L)	309	28	10–40
Alanine aminotransferase (U/L)	292	32	7–56
Alkaline phosphatase (U/L)	61	53	40–120
Gamma-glutamyl transferase (U/L)	25	21	10–60
Serum albumin (g/dL)	3.5	3.6	3.5–5.0
Serum sodium (mmol/L)	134	138	135–145
Serum potassium (mmol/L)	3.5	4	3.5–5.0
Serum total calcium (mmol/L)	1.8	1.9	2.1–2.6
Serum magnesium (mmol/L)	0.8	0.9	0.7–0.95
Serum phosphate (mmol/L)	1.8	1	0.8–1.5
Electrocardiography	Sinus rhythm		
Troponin I (ng/L)	2,741	5	<16
Creatine phosphokinase (U/L)	1,254	90	30–135
Urine full report			
Appearance	Clear	N/A	Clear
Pus cells (/HPF)	99	N/A	0–5
Red blood cells (/HPF)	55	N/A	0–2
Protein	Trace	N/A	Nil
Sugar	Negative	N/A	Negative
Fasting blood sugar (mg/dL)	90	N/A	70–99
Serum ferritin (ng/mL)	2,200	N/A	15–150
Blood/Urine/Stool cultures	Blood culture: MSSA isolated; other blood cultures negative/contaminated urine and stool culture negative		
Cerebrospinal fluid analysis			
Color	Colorless	N/A	Colorless
Protein (mg/dL)	41	N/A	5–45
Glucose (mg/dL)	55	N/A	45–80
Polymorphs (cells/µL)	0	N/A	0
Lymphocytes (cells/µL)	5	N/A	0–5
Red blood cells (cells/µL)	330	N/A	0
Adenosine deaminase (U/L)	2	N/A	0.67–2.07
CSF TB Gene-pert	Negative	N/A	Negative
Anti-nuclear Antibody	Negative	N/A	Negative
Anti-neutrophil cytoplasmic antibodies	Negative	N/A	Negative
Infective screening			
Toxocariasis	Negative	N/A	Negative
Cryptococcal antigen	Negative	N/ A	Negative
Melioidosis antibody	Negative	N/A	Negative
Rickettsia serology: *Orientia tsutsugamushi* IgG (scrub typhus)	Negative	N/A	Negative
*Rickettsia conorii *IgG	Negative	N/A	Negative
Brucellosis Ab	Negative	N/A	Negative
Hepatitis B/C	Negative	N/A	Negative
Retroviral studies	Negative	N/A	Negative
Malaria	Negative	N/A	Negative

**Figure 5:**
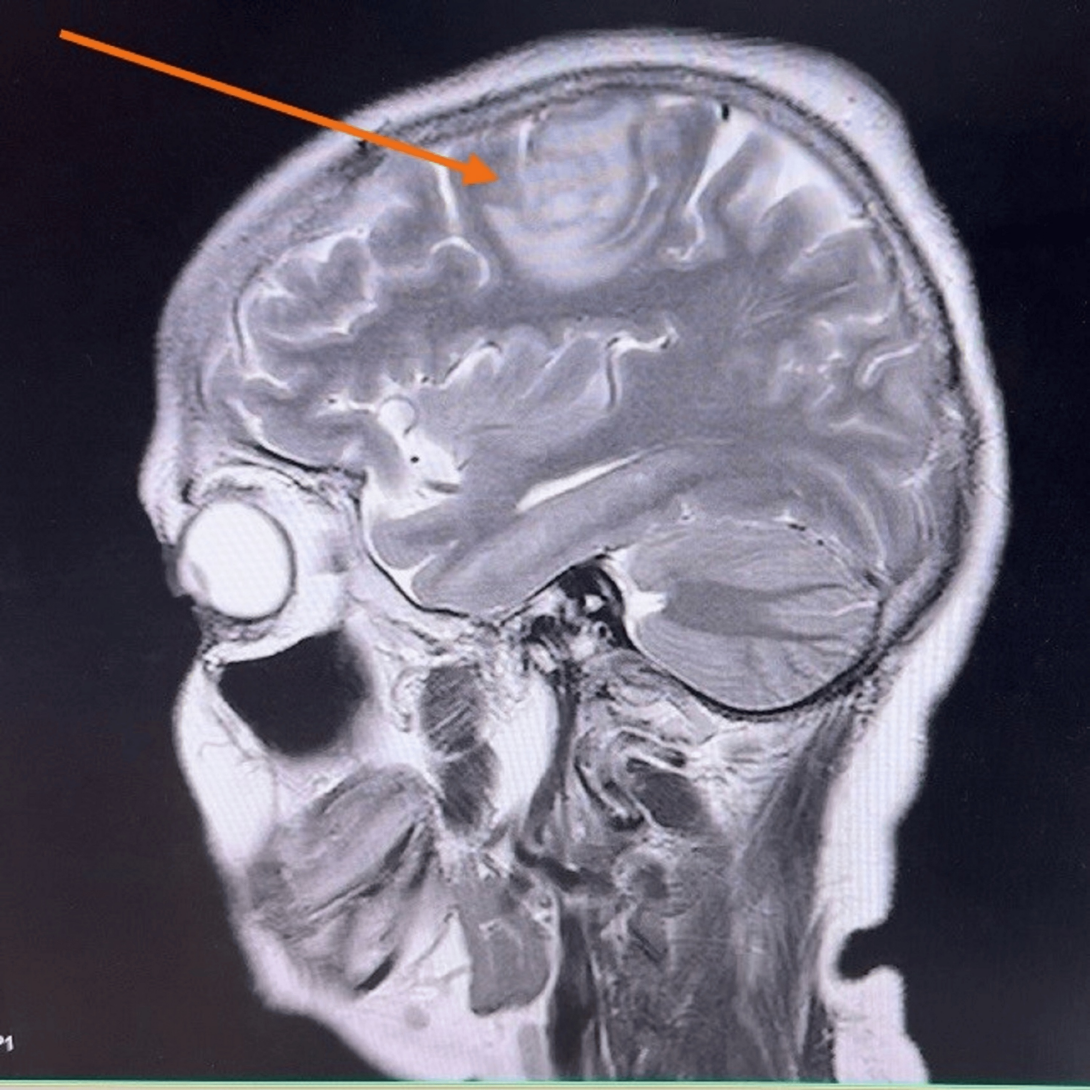
Coronal section of the patient’s brain contrast MRI showing brain abscess in left frontal lobe (24 mm × 15 mm × 17 mm) (red arrow) and a further small area of early abscess formation in the right posterior temporal lobe.

**Figure 6:**
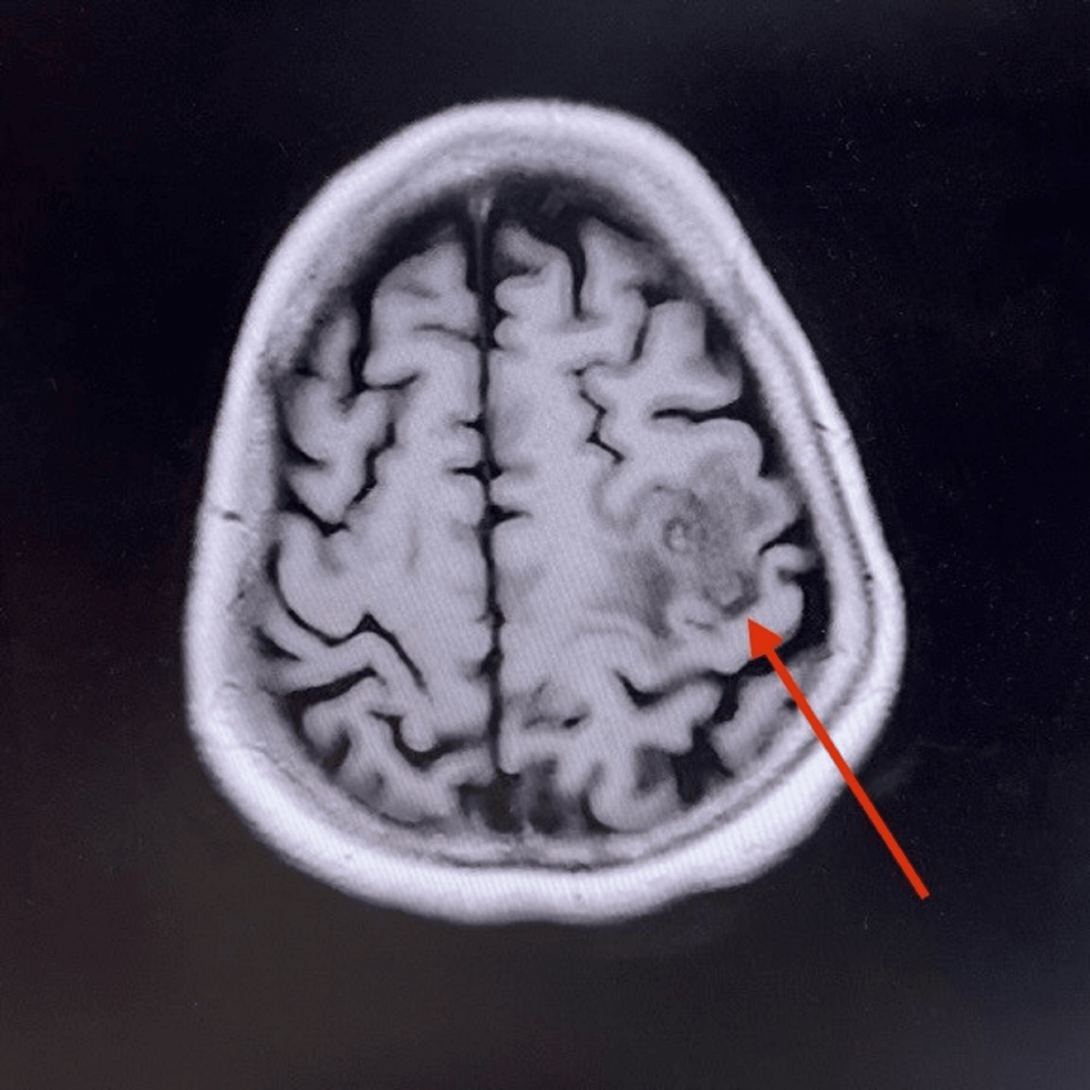
Sagittal section of the patient’s brain contrast MRI showing brain abscess in left frontal lobe (24 mm × 15 mm × 17 mm) (red arrow) and a further small area of early abscess formation in the right posterior temporal lobe.

Table 2 summarizes the imaging and procedural findings. Figure 5 and Figure 6 show coronal and sagittal contrast-enhanced MRI of the patient’s brain, demonstrating cerebral abscesses consistent with septic embolization.

**Table 2:** Imaging and procedural investigations.

Investigations	Findings
Chest X-ray	Bilateral reticular nodular shadows; left lower zone opacity
High-resolution CT chest	Normal
Ultrasound abdomen	L/pyelonephritis
Non-contrast CT brain	Left frontal hypodense area noted
MRI brain	Early cerebral abscess in the left frontal lobe; small lesion in the right temporal region (Figures 5, 6)
Transthoracic echocardiogram	7 mm oscillating mass on the posterior mitral valve leaflet; mild mitral regurgitation
Transesophageal echocardiogram	Hyperechogenic oscillating lesion (0.7 × 0.5 cm) on the posterior mitral valve leaflet — vegetation
Follow-up transesophageal echocardiogram after treatment	No vegetations; normal mitral valve; no abscess

The likely source of MSSA bacteremia was identified as multiple septic teeth, which were removed by the Oral and Maxillofacial Surgery team. A multidisciplinary approach was adopted involving cardiology, neurology, microbiology, nephrology, and infectious diseases. She was given intravenous antibiotic therapy for six weeks via a central venous line. The neurosurgical team recommended purely medical management for the brain abscesses. Supportive management for renal impairment and electrolyte abnormalities was provided. No dialysis was required.

After six weeks of intravenous antibiotics, the patient became afebrile and clinically stable. Inflammatory markers and renal function progressively improved. Follow-up transesophageal echocardiogram showed complete resolution of vegetation. Repeat MRI demonstrated an interval reduction of abscess size. She was discharged with scheduled outpatient follow-up and remained clinically well at her two-week review, with improved appetite and no neurological symptoms.

## Discussion

IE is an infection (acute or subacute) affecting the cardiac endothelium, mainly the heart valves. It has a high mortality rate, reaching up to one-third of the affected patients. Early disease recognition and proper early treatment are essential to prevent the disease-related complications and mortality [1]. There is a wide range of infectious etiologies causing IE. Poor oral hygiene and multiple infected teeth likely served as the portal of entry in this case, consistent with the well-established association between dental sepsis and left-sided endocarditis.

IE caused by *S. aureus* remains one of the most aggressive forms of bacterial endocardial infection and is frequently associated with multisystem complications. MSSA IE typically presents with a rapid onset of high-grade fever and systemic inflammatory manifestations. In this patient, classical peripheral stigmata, including splinter hemorrhage, Janeway lesions, and Osler’s nodes, were prominent, supporting an embolic and immunological component of the disease. Our patient fulfilled the modified Duke criteria, which is currently widely used for clinical diagnosis of IE with clinical, laboratory, and echocardiographic findings [2].

According to studies, the risk of septic embolization in IE depends on several factors, including the vegetation’s size, motility of the vegetation, location on the mitral valve, and staphylococcal bacteremia [3]. IE associated with a central nervous system event is one of the common complications and is associated with high mortality and an important factor determining prognosis [4]. Neurological complications occur in approximately 20-40% of *S. aureus* IE cases, with stroke being the most common. Patients can also develop transient ischemic attack due to vaso-occlusion, cerebral abscesses, mycotic aneurysms, and intracranial and subarachnoid hemorrhages as neurological complications [5]. Brain abscess is more frequently a feature of acute endocarditis than subacute endocarditis. The abscesses may be single or multiple, and their clinical presentation may be that of a space-occupying lesion, encephalopathy, or with features of meningitis [6]. Our patient demonstrated two abscesses in the frontal and temporal regions, an expected distribution given hematogenous spread. MRI remains the most sensitive modality for identifying early abscess formation, where brain abscesses are seen as multiple ring-enhancing lesions at the gray-white matter junction.

Glomerulonephritis associated with IE can result from multiple mechanisms, including drug toxicity, septic emboli, or immune-complex glomerulonephritis. It has a broad spectrum and is relatively rare, especially in cases with no previous heart disease [7]. The patient’s acute kidney injury, accompanied by proteinuria and microscopic hematuria, aligned most closely with infection-related glomerulonephritis. This is consistent with elevated inflammatory markers, presence of a high systemic bacterial load, and the absence of features suggestive of primary vasculitis or direct septic embolization. *S. aureus*-associated infection-related glomerulonephritis is increasingly recognized and may present with severe but reversible renal dysfunction, as demonstrated by gradual improvement following withdrawal of nephrotoxic agents and infection control.

This patient was managed by a multidisciplinary team comprising cardiology, neurology, neurosurgery, microbiology, nephrology, and oral maxillofacial surgery, as recommended by the latest guidelines that emphasize coordinated care for complex IE presentations. The duration of intravenous antibiotics is six to eight weeks, along with serial monitoring of clinical and biochemical parameters and serial imaging to assess the size of the cerebral abscess and vegetation in the heart to guide the treatment.

## Conclusions

Overall, this case emphasizes the classical yet challenging natural history of *S. aureus* IE with multisystem involvement, as in our case, cerebral abscesses and infection-related glomerulonephritis. It reinforces the importance of early recognition of peripheral stigmata, the role of thorough multisystem evaluation, actively looking for the infectious source, and the importance of serial imaging and laboratory monitoring. It also highlights that severe complications, such as cerebral abscesses and infection-related glomerulonephritis, can be treated successfully with timely diagnosis and targeted antimicrobial therapy with multidisciplinary management.
